# Testing the treatment effect on competing causes of death in oncology clinical trials

**DOI:** 10.1186/1471-2288-14-72

**Published:** 2014-05-29

**Authors:** Federico Rotolo, Stefan Michiels

**Affiliations:** 1 Gustave Roussy, Service de Biostatistique et d’Épidémiologie, F-94805 Villejuif, France; 2 Univérsité Paris-Sud, F-94805 Villejuif, France

**Keywords:** Competing risks, Peto’s test, Cause of death, Cancer death, Cumulative incidence function, Cause-specific hazard, Gray’s test

## Abstract

**Background:**

Chemotherapy is expected to reduce cancer deaths (CD), while possibly being harmful in terms of non-cancer deaths (NCD) because of toxicity. Peto’s log-rank test is popular in the medical literature, but its operating characteristics are barely known. We compared this test to the most common ones in the statistical literature: the cause-specific hazard test and Gray’s test on the hazard of the subdistribution. We investigated for the first time the impact of reclassifications of causes of death (CoD) after recurrences, and of misclassification of CoD.

**Methods:**

We present a simulation study in which we varied the censoring rate and the correlation between CD and NCD times, we generated recurrence times to study the role of the reclassification of CoD, and we added 20% misclassified CoD. We considered four scenarios for the treatment effect: none; none for CD and negative for NCD; positive for CD and none for NCD; positive for CD and negative for NCD. We applied the three tests to a randomized clinical trial evaluating adjuvant chemotherapy in 1,867 patients with non-small-cell lung cancer.

**Results:**

Most often the three tests well preserved their nominal size, Gray’s test did not when the treatment had an effect on the competing CoD. With a high rate of misclassified CoD, Gray’s and the cause-specific tests lost much of their power, whereas the Peto’s test had the highest power. The cause-specific test had inflated size for NCD when the treatment was beneficial for CD with many misclassified CoD, but had the highest power for NCD when the treatment had no effect on CD, and had similar power to Peto’s test for CD when the treatment had no effect on NCD. Gray’s test performed best when the effect on the two CoD was opposite. The higher the censoring, the lower the rejection probabilities of all the tests and the smaller their differences.

**Conclusions:**

In this first head-to-head comparison of the three tests, the cause-specific test often proved to be the most reliable. Comparing results with and without misclassification of the CoD, Peto’s test was the least influenced by the presence of such misclassification.

## Background

The analysis of survival data in the presence of competing risks has been a widely debated topic for many decades in both the statistical [1-5] and the medical literature [6-11]. Interest in the subject gained momentum in the 1990s, when two main approaches emerged: an approach based on the cause-specific hazard function and another based on the cumulative incidence function and its associated hazard of the subdistribution. For a detailed discussion see [12] for example. An issue of particular importance in clinical research is testing the effect of covariates – typically the treatment – on competing causes of death. Different solutions have been proposed. The most common ones in the statistical literature are the log-rank test for the cause-specific hazard [1,13] and nonparametric and semiparametric tests for the cumulative incidence function (CIF) [14,15]. However, Peto and the Early Breast Cancer Trialists’ Collaborative Group proposed the log-rank subtraction method in the context of oncology [16-19], which is quite popular in the clinical literature and especially in meta-analyses: see for instance references [20-24]. This test imputes deaths to the cancer whenever the cause is unknown or when they occur after a recurrence, whatever the recorded cause. It calculates cause-specific mortality as the difference between overall mortality and that attributable to other causes. The authors assert that this approach makes the test unbiased for the assessment of the effect on cancer mortality.

The comparison of different methods from a theoretical point of view and via simulation studies is being considered with increasing interest in the literature. Putter et al. [3] offered a detailed and insightful review of competing risks methodology. Dignam et al. [10] and Dignam and Kocherginsky [25] focused on point estimation of the treatment effect according to different modeling approaches. Pintilie [26] provided a simulation study, with independent variables of the times to death by cause, showing that the tests based on the cause-specific hazard – Wald, score and likelihood-ratio – have the correct size and power, in the absence of any effect on the competing event. Using simulations, Freidlin and Korn [27] compared the cause-specific log-rank test to Gray’s non-parametric test [14] for the CIF. They concluded that the former preserves its nominal size better and has greater power than the latter, even with positively correlated event times. Williamson et al. [28] extended these results showing that Gray’s test has greater power in the case of very different degrees of negative correlation between competing event times in the two treatment arms. Ruan and Gray [29] studied Peto’s test both analytically and in simulations with independent survival times. They proved that it has good properties when the rates of competing events are similar, whereas it has an inflated size and poor power otherwise.

For the first time we present in this article a simulation study to compare head-to-head Peto’s log-rank subtraction test to the log-rank test on the cause-specific hazard, and to Gray’s test based on the CIF in a broad set of clinical scenarios. In order to investigate the effect of different classifications of the cause of death established by Peto’s test, we used a simulation method that allows relapse times to be generated in addition to cancer-death (CD) and non-cancer-death (NCD) times. We simulated data with negative, null, and positive correlations, thereby covering an exhaustive range of dependence assumptions. This study is the first which investigates the impact of censoring and, most importantly, of misclassification of causes of death on the behaviour of these tests.

The clinical problem motivating this study was the evaluation of the efficacy of adjuvant chemotherapy for patients with non-small-cell lung cancer in the International Adjuvant Lung Cancer Trial (IALT) [30,31]. Its interest is to test whether chemotherapy has a beneficial effect on the occurrence of CD, taking into account the fact that patients can meanwhile die of other causes, and that an increased risk of NCD is possible in the treatment arm, due to chemotherapy toxicity.

In the next section, we present the test statistics of interest. Then, we provide details on the simulation study and its results. Finally, we present the IALT study and the results of the tests for CD and NCD.

## Methods

### Tests for competing causes of death

There are different approaches to dealing with duration data in the presence of competing events, such as cause-specific death and death from other causes. In a latent failure time perspective, there is a random variable *T*_*i*_ for the time to each possible event, but only the time to the first event can be recorded. The hazard function of the marginal distribution of each *T*_*i*_ is usually called the cause-specific hazard. Testing the treatment effect on the cause-specific hazard allows one to evaluate the net effect of covariates on each event; even though quite intuitive, this strategy has been criticized because it compares the treatment arms in terms of the risk of each event type while ignoring all the others. Gray [14] proposed an alternative approach, based on the CIF, which takes into account all types of events. As the hazard of the CIF incorporates information on all the competing risks, testing the effect of the treatment on the incidence of each type of event also reflects its effect on all the others. As variations of the risk of each event reverberate on the hazards of competitors, it is advised to consider their results in combination with the analysis of all cause-specific hazards [4]. Moreover, due to its mathematical definition, the hazard of the CIF requires that patients who experience an event remain in the risk sets of the other types of event. For a detailed discussion of this topic, we refer to Section 3 of [3] and Chapters 4 to 6 of [12].

Consequently, there are also several approaches for testing the effect of a treatment on competing events. We restricted ourselves to considering three of the most popular ones: the cause-specific and the Gray tests, which receive most of the attention through methodological research, and the Peto test, which is quite common in the medical literature. We aim to compare them in several clinically relevant situations.

#### ***Peto (*** ***Pe*** ***)***

The log-rank subtraction test proposed and further described by Peto [16,17] consists in a piecewise (with respect to time) version of the log-rank test, performed separately by cause of death. It is said to be a subtraction method because the quantities used to compute the test statistic are first calculated for overall mortality and for NCD. Those concerning CD are then obtained by taking the difference between the former two. Another relevant peculiarity of this approach is that all deaths due to an unknown cause and all those occurring after a relapse are ascribed to the cancer, even if explicitly declared as due to another cause.

#### ***Cause-specific (*** ***CS*** ***)***

Historically, the simplest and most naive approach adopted reflects the idea of considering only the relevant events for each cause of death, while treating all the competing events as independent censoring. This leads to the use of the log-rank test on the cause-specific hazard [1,13], which is approximately equivalent to the score test of the cause-specific Cox model which itself is asymptotically equivalent to the Wald and likelihood-ratio tests in the same model.

#### ***Gray (*** ***Gr*** ***)***

Another popular approach in the context of competing risks is the one based on the CIF, for which the assumption of independence of the competing events is not required. The hazard associated with the CIF, called the hazard of the subdistribution, also takes into account the occurrence of competing events. In particular, when a subject experiences a competing event, his/her time to the relevant event is not censored and he/she remains in the risk set. Gray’s nonparametric test [14], used in our study, is asymptotically equivalent to the Wald test on the regression parameter in the Cox model of the hazard of the subdistribution [15] when there is no censoring.

### Plots

In the example presented later on, we will show the cumulative risk and incidence curves for all, non-cancer and cancer deaths by treatment arm. They will be plotted by means of three methods corresponding to the three tests for the treatment effect. The first, corresponding to the cause-specific test, is the Nelson–Aalen method for the (cause-specific) cumulative risk [32,33]. In the case of cause-specific risks, only deaths declared due to the cause of interest are considered as events by the Nelson–Aalen estimator, while all other deaths are censored (assuming non informative censoring). The plots in the second group are the Peto estimator of the (cause-specific) cumulative yearly rates; in these plots all deaths following a recurrence are classified as CD, as well as those of an unknown cause. NCDs preceded by a recurrence are censored when a recurrence occurs. According to the Peto method, first the survival probability is computed per year for the 2 arms combined. Then the survival probability for each arm is obtained by adding to it or subtracting from it a quantity which depends on the logarithm of the yearly risk ratio [16,17]. Finally, the Aalen–Johansen estimates of the CIFs [34], corresponding to the Gray test, are plotted. It is noteworthy that, in the case of overall survival, one minus the CIF corresponds to the Kaplan–Meier estimate.

### Simulation study

Testing the efficacy of the therapy on the time to CD and NCD is the focus of the researcher’s interest. Sometimes, the classification of causes of deaths implicitly requires the occurrence of a recurrence (Rec). Although the treatment evaluation is done directly on times to CD and NCD, the tests differ in the manner of classifying causes of death. In particular, the Peto test requires information on the times to recurrence.

**Figure 1:**
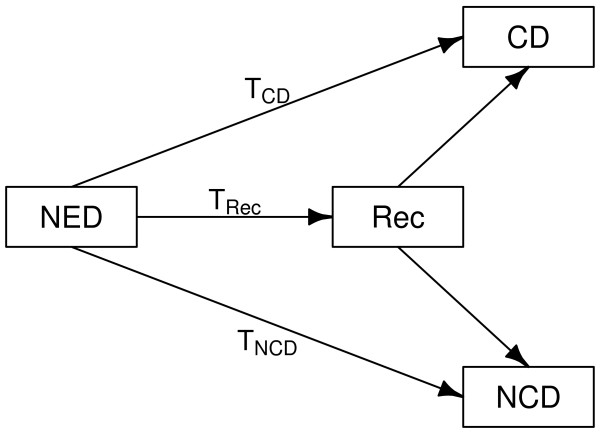
**Event history structure used for data simulation. **T: time to different events. NED: no evidence of disease after initial treatment. Rec: recurrence; CD: cancer death; NCD: non-cancer death.

We first considered the times to CD and NCD (Figure 1). We generated them by using two exponential distributions, possibly with positive or negative dependence. We obtained them in two steps. First, a bivariate normal random variable *Z *= (*Z*_1_,*Z*_2_)^⊤^ was generated with unit means, unit variances and correlation *ρ*. Then, the times to death were computed as *T*_CD _= - log(*Φ *(*Z*_1_))/*λ*_CD_ and *T*_NCD _= - log(*Φ*(*Z*_2_))/*λ*_NCD_, where *Φ *(·) is the standard normal distribution function [27]. Thus, *T*_CD _∼ *Exp *(*λ*_CD_) and *T*_NCD _∼ *Exp *(*λ*_NCD_). In the control group of the IALT trial, which we describe below, we estimated that the CD rate is about five-fold higher than the NCD rate. Therefore, we set $\lambda_{\text{CD}}=\sqrt{5}≃2.24$ and $\lambda_{\text{NCD}}=1/\sqrt{5}≃0.45$. The time to death for each subject is then *T*_*D *_= min(*T*_CD_,*T*_NCD_). Finally, we assumed that, conditional on the time to CD, the time to recurrence *T*_*Rec *_ follows a uniform distribution between 0 and *T*_CD_. Hence, a recurrence is observed whenever *T*_*Rec *_< *T*_*D *_and is censored only when *T*_*Rec *_> *T*_NCD_. This method allowed us to study the effect of the reclassification done by Peto: in our simulations about half of the NCD were preceded by a recurrence. We did not consider the case of unknown causes of death, which were very marginal in our real dataset.

Here we present different scenarios concerning the treatment effect. Figure A.1 in the Additional file 1 shows, for the first scenario, the correlations obtained between the event times, depending on *ρ*, the correlation of the underlying normal random variables: the relation is roughly linear and setting the parameter *ρ* can be considered almost equivalent to setting the correlation between CD and NCD times. On the other hand, this does not affect the correlation between CD and recurrence times, which can be shown to be constantly $\sqrt{3/5}=0.77$. In this respect, no difference exists between the scenarios. In order to investigate the properties of the tests in a wide range of situations, we chose five values for *ρ*, covering very negative and very positive dependence, passing through weak and no dependence: -0.75, -0.375, 0, 0.375, 0.75.

We examined four clinical situations for the effect of the treatment on the occurrence of death from cancer and from other causes: 

1. a null effect on both CD and NCD (*HR*_CD _= *HR*_NCD _= 1),

2. a null effect on CD (*HR*_CD _= 1) and an increased NCD risk (*HR*_NCD _= 1.25),

3. a reduction of the risk of CD (*HR*_CD _= 0.8) and a null effect on NCD (*HR*_NCD _= 1),

4. a reduction of the risk of CD (*HR*_CD _= 0.8) and an increased NCD risk (*HR*_NCD _= 1.25).

**Figure 2:**
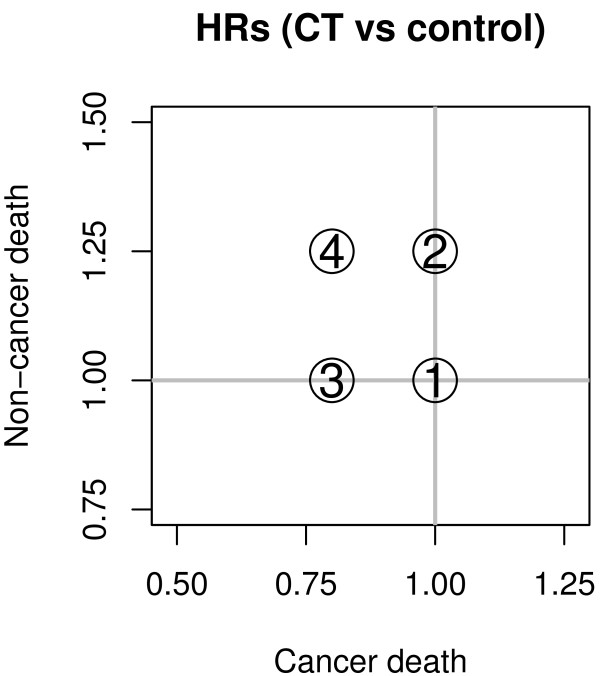
**Hazard ratios in the simulation study.** Hazard ratios for cancer death and non-cancer death in the four scenarios used for the simulation study.

The first scenario is the complete null scenario, i.e. the one in which both the null hypotheses of no treatment effect are true. The second is the most pessimistic, where the treatment is toxic and ineffective. The third scenario is the ideal target for a treatment in oncology, which just reduces the risk of CD. Finally, the fourth one is a scenario that could occur for chemotherapy and radiotherapy regimens in oncology, as their efficacy against CD implies a cost in terms of an increased NCD hazard. The hazard ratios for the treatment effect in the four scenarios are illustrated in Figure 2. In addition to the situation with complete data, we replicated simulations with 25% and 50% of censored observations. Censoring times were generated from uniform random variables between zero and a given bound. For each scenario, the choice of this upper bound was made numerically in order to attain the desired proportion of censored times to death. As in clinical practice the causes of death can be misrecorded, we also reperformed all the tests after inverting the cause (CD *vs. *NCD) of 20% of deaths.

### The International Adjuvant Lung Cancer Trial

The IALT recruited 1,867 patients who underwent complete surgical resection of non-small-cell lung cancers. They were randomly assigned to cisplatin-based adjuvant chemotherapy (932) or the control (935) group and were followed up for 10 years (median: 7.5 years). The International Adjuvant Lung Cancer Trial Collaborative Group [30] and Arriagada et al. [31] showed that adjuvant chemotherapy provides a benefit in terms of both overall and disease-free survival at 5 years. As shown in Table 1, 1,168 (62.6%) out of 1,867 patients died during follow-up, 578 in the experimental and 590 in the control arm. Among all the recorded deaths, 918 (78.6%) were ascribed to lung cancer, 179 (15.3%) to other causes, and 71 (6.1%) to unknown causes. Among the 197 patients who died of non-cancer causes, 71 had a recurrence recorded; among the 71 patients who died of unknown causes, 26 had a recurrence recorded. In total, 97 deaths that occurred after a recurrence and declared due to non-cancer or unknown causes were reclassified as due to the cancer by the Peto method.

**Table 1:** Causes of deaths by treatment arm in the IALT study

	**Chemotherapy**	**Control**	**Total**	
Cancer Deaths	438	480	918	
Non-Dancer Deaths	107	72	179	(Of which 71 after a relapse)
Deaths from unknown cause	33	38	71	(Of which 26 after a relapse)
All Deaths	578	590	1168	

The Ethics Committee of Kremlin-Bicêtre hospital in France France (Comité de Protection des Personnes Île-de-France VII) approved the protocol on January 9, 1995. When the study began in 1995, informed consent was obtained from each patient according to the regulations of the participating country; in 1999, all participants were required to give written informed consent.

## Results and discussion

As described above, we considered four scenarios for the treatment effect, five possible degrees of dependence between the times to CD and NCD, three possible proportions of censoring, and presence or absence of misclassification of the cause of death. For each of these 4 × 5 × 3 × 2 = 120 situations, 10 000 data sets of size 1000 were generated. The three tests were performed for each of them and the empirical rejection probabilities at a 5% nominal size were computed across the 10 000 replications. The null hypothesis of no treatment effect holds in scenarios 1 and 2 for CD and in scenarios 1 and 3 for NCD. In these cases the empirical rejection probabilities stand for the empirical size of the tests. On the contrary, in all the other situations, the hypothesis does not hold and the rejection probabilities represent the empirical power of the tests. Of note, the rate of miclassified causes of death (20%) is quite high with respect to clinical real life, but it is useful in this context to study its role in a somehow extreme situation.

In the null scenario, i.e. in the absence of any treatment effect on both causes of death, all the tests have empirical rejection probabilities that are very close to the nominal size of 5% (range: 0.04 – 0.06; Additional file 1: Table A.1 and Figure A.2) and their use is equivalent. Furthermore, none of censoring, correlation between causes of death, and misclassification of causes of death (Additional file 1: Table A.2 and Figure A.3) affect the results.

**Figure 3:**
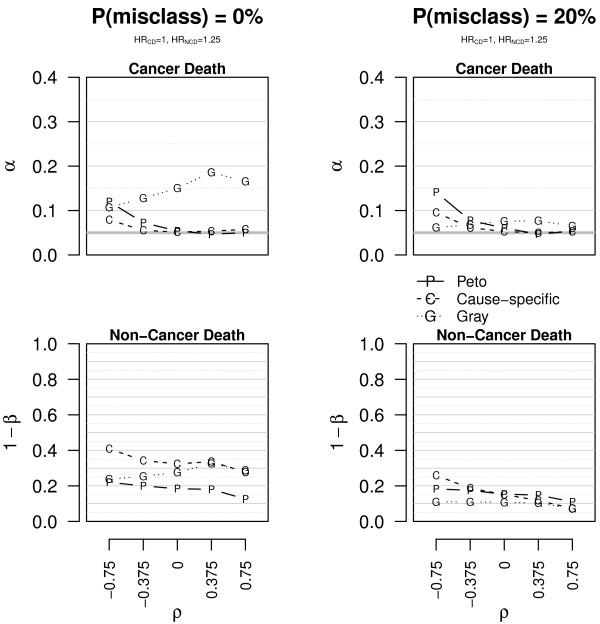
**Empirical size and power of the tests in scenario 2.** Empirical size (*α*) and power (1 - *β*) of the tests for CD and NCD in scenario 2 (*HR*_CD _= 1, *HR*_NCD _= 1.25). The data are simulated without censored observations. The plots on the first line concern CD, those on the second line NCD. The plots on the left are for data with correct causes of death **(P(misclass) = 0%)**, those on the right for data with 20% of misclassified causes of death **(P(misclass) = 20%)**. The bold grey horizontal line corresponds to the 0.05 level and *ρ* to the correlation.

In the second scenario, we considered the case where the therapy is not effective for reducing CD, but it is harmful in terms of NCD, because of toxicity. Figure 3 shows the main results with complete data, whereas full details with 25% and 50% censored observations are provided in Additional file 1: Table A.3 and Figure A.4. Let’s first consider the results when there is no misclassification of the cause of death. Under these conditions results show that for complete data Gr (Gray test) has an overinflated size for CD (0.10 < *α *< 0.19, complete data), whereas the other two tests have better empirical sizes in general (0.04 < *α *≤ 0.12 for Pe [Peto test] and 0.05 < *α *< 0.08 for CS [Cause-Specific test], complete data). Due to the set-up of our simulation study with a CD rate about 5-fold higher than a NCD rate, the three tests have moderate power for detecting an effect for NCD (0.12 < 1 - *β *< 0.41, complete data), with CS outperforming its two competitors and Pe being the least powerful (1 - *β *< 0.23). As censoring increases, all the rejection probabilities decrease in general and get closer and closer to each other, so that the differences between them become less and less pronounced. CS seems to be the most reliable choice in this context. In the case that 20% of the causes of death are misrecorded (see also Additional file 1: Figure A.5 and Table A.4), the size of Gr is more correct (*α *∈ [ 0.06,0.08], complete data) and the three tests loose power for detecting the effect on NCD, notably CS (1 - *β *< 0.26) and Gr (1 - *β *< 0.13).

**Figure 4:**
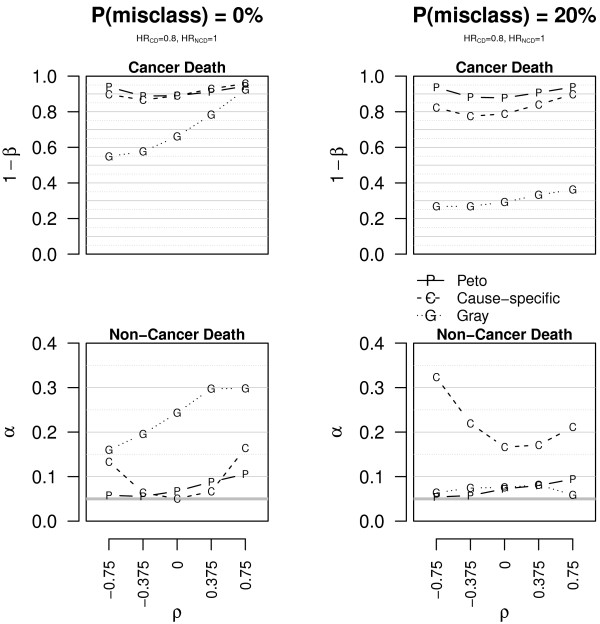
**Empirical power and size of the tests in scenario 3.** Empirical power (1 - *β*) and size (*α*) of the tests for CD and NCD in scenario 3 (*HR*_CD _= 0.8, *HR*_NCD _= 1). The data are simulated without censored observations. The plots on the first line concern CD, those on the second line NCD. The plots on the left are for data with correct causes of death **(P(misclass) = 0%)**, those on the right for data with 20% of misclassified causes of death **(P(misclass) = 20%)**. The bold grey horizontal line corresponds to the 0.05 level and *ρ* to the correlation.

Scenario 3 represents the target situation for a cancer treatment that is just effective on CD, without any effect on NCD. Under these conditions and without misclassified causes of death, the results in Figure 4 (see also Additional file 1: Figure A.6 and Table A.5) suggest that Gr has the lowest power for CD (0.54 < 1- *β *< 0.93 for Gr, while 0.86 < 1 - *β* for Pe and CS; complete data) and often by far the highest size for NCD (0.16 < *α*; complete data). CS and Pe are largely equivalent for CD. Either CS or Pe is preferable for NCD (0.05 < *α *< 0.17 for CS, 0.05 < *α *< 0.11 for Pe; complete data), depending on the correlation. Again, censoring causes a contraction of the empirical rejection probabilities, irrespective of whether the null hypothesis holds or not. In this scenario Pe and CS are broadly equivalent, whereas Gr should not be preferred. When introducing miclassification of the cause of 20% of deaths (see also Additional file 1: Figure A.7 and Table A.6), CS is less powerful for CD (0.77 < 1 - *β*; complete data) and has very inflated size for NCD (0.10 < *α *< 0.33); Gr has very poor power for CD (1 - *β *< 0.37, complete data) but is more correct for NCD (0.04 < *α *< 0.09); again, Pe is less sensitive to misclassification as it reclassifies at least some of the deaths as due to the cancer when a recurrence occurs, irrespective of the declared cause.

**Figure 5:**
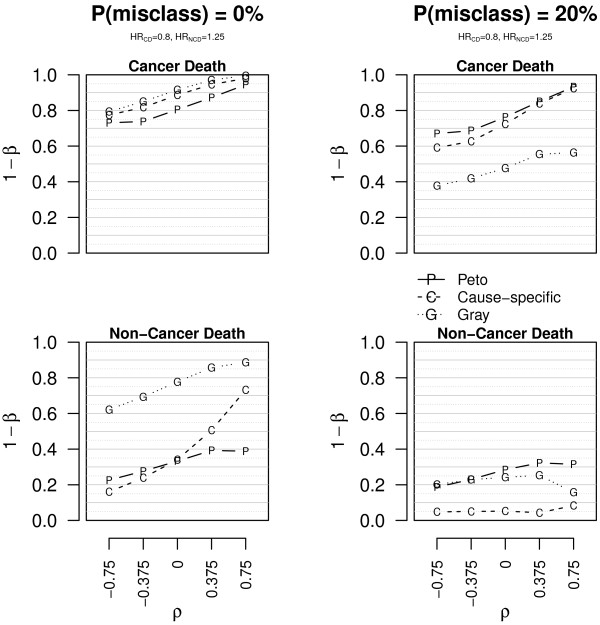
**Empirical power of the tests in scenario 4.** Empirical power (1 - *β*) of the tests for CD and NCD in scenario 4 (*HR*_CD _= 0.8, *HR*_NCD _= 1.25). The data are simulated without censored observations. The plots on the first line concern CD, those on the second line NCD. The plots on the left are for data with correct causes of death **(P(misclass) = 0%)**, those on the right for data with 20% of misclassified causes of death **(P(misclass) = 20%)**.

Finally, Figure 5 (see also Additional file 1: Figure A.8 and Table A.7) provides empirical powers if the treatment has a beneficial effect on the risk of CD, but at a cost of a harm in terms of NCD hazard. Gr is uniformly the most powerful in this scenario. In particular, for NCD it is in general 35–40% more powerful than its competitors (0.62 < 1 - *β *< 0.89 for Gr, 0.16 < 1- *β *< 0.74 for CS and 0.22 < 1 - *β *< 0.40 for Pe; complete data). The rejection probabilities are far more similar for CD, with high power ranging from 0.73 to 1.00 for all tests (complete data). In all the scenarios, the tests are generally more powerful for CD than for NCD because the baseline hazard for CD is considerably higher than for NCD (*λ*_CD _= 5 × *λ*_NCD_). Even though censoring attenuates differences between the three tests, Gr is undoubtedly preferable under these conditions. On the other hand, Gr has the highest loss of power due to misclassification of the cause of death (see also Additional file 1: Figure A.9 and Table A.8) notably for CD (1 - *β *< 0.57, complete data); for NCD the widest power loss is for CS (1 - *β *< 0.09).

**Figure 6:**
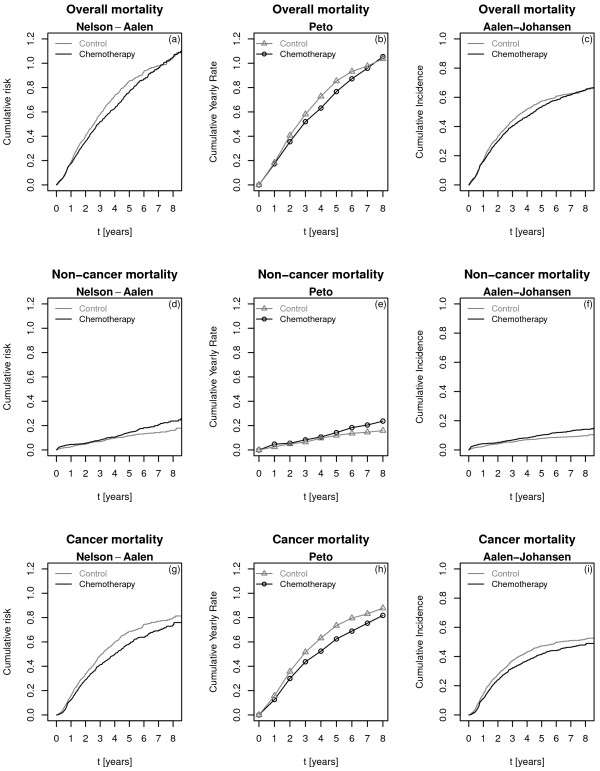
**Overall and cause-specific mortality for control and treatment arms in the IALT trial. ** First column **((a), (d), (g))**: Nelson – Aalen estimates of the cumulative hazards. Second column **((b), (e), (h))**: Peto estimates of the cumulative yearly rates. Third column **((c), (f), (i))**: Aalen - Johansen estimates of the cumulative incidence functions. First line **((a) – (c))**: overall mortality. Second line **((d) – (f))**: non-cancer mortality. Third line **((g) – (i))**: cancer mortality.

In the International Adjuvant Lung Cancer Trial, the separate evaluation of the chemotherapy effect on the risks of CD and NCD is of primary interest. Plots on the first line of Figure 6 show the Nelson–Aalen estimate of the cumulative risk (a), the cumulative yearly rates estimated by the Peto method (b), and the cumulative incidence function (c), respectively, for overall mortality by treatment arm. Note that, as no competing event exists for overall survival, plot 6(c) corresponds to one minus the Kaplan-Meier estimate. Chemotherapy seems to provide a benefit up to five years after randomization, and then the two curves overlap. Under a proportional hazards assumption, the estimated hazard ratio between the chemotherapy and the control groups is 0.95 (95% CI: 0.84-1.06) and the log-rank test has a p-value equal to 0.34. Note that, for the sake of simplicity, we did not adjust for any of the prognostic factors used in previous publications about the IALT study. The desired and expected action of cisplatin-based chemotherapy is to reduce the risk of CD, while having no effect or moderately increasing the risk of NCD. Figures 6(g) – 6(i) show the same quantities as (a)–(c) but only for CD; you can see that risk and incidence are constantly less in the chemotherapy group than in the control group. On the other hand, Figures 6(d) – 6(f) show that the two treatment arms are overall equivalent with respect to non-cancer mortality; an increased NCD rate and incidence are observed for the experimental group after five years. Then, we compared the results of testing the effect of chemotherapy on the competing causes of death by means of the three test statistics considered thus far: Pe, CS and Gr (Table 2). The increase observed in NCD in the treatment arm (see Figure 6(d)) is significant according to the three tests: *p *= 0.029 for Pe, *p *= 0.041 for CS and *p *= 0.015 for Gr. One should keep in mind that the Pe reclassifies as CD a total of 97 deaths: 26 NCDs – which could attenuate the differences between treatment arms – and 71 deaths from an unknown cause. These deaths from an unknown cause are censored for both causes of death by CS, whilst they make up a third group according to Gr.

**Table 2:** **Results of the three tests for the treatment effect on **CD** and **NCD**, in the IALT study**

	CD		NCD	
	***X***^**2**^	**(p – val)**	***X***^**2**^	**(p – val)**
Pe	3.72	(0.054)	4.77	(0.029)
CS	3.44	(0.064)	4.19	(0.041)
Gr	4.52	(0.033)	5.89	(0.015)

The values of the test statistics (*X*^2^) are provided together with the associated p-values (p–val).

The difference in survival in favor of the treatment arm, which is non-significant for overall survival, is significant or borderline for CD, with p-values ranging from 0.033 to 0.064. This suggests that the effects on the risks of CD and NCD are in opposite directions, and that they compensate each other, at least partially, when all deaths due to any cause are considered together. Gr, based on the CIF, detects a statistically significant difference at a 5% level (*p *= 0.033), whereas the other two are borderline but not significant (*p *= 0.054 for Pe, *p *= 0.064 for CS). Most likely, the net increase (i.e. in the cause-specific hazard) in the risk of NCD in the chemotherapy arm contributes to reducing the incidence of CD in that group, amplifying the reduction in the risk of CD when measured in terms of the CIF, although the differences between the test statistics are small.

Both the CS and the Pe tests treat death from other causes as independent censoring, which is not realistic in most practical situations. Gr does not require such an assumption but on the other hand its estimated effect on each competing event reflects also the effect on the others. Thus, both the approaches have a possible drawback, but none of the two prevailed clearly in the simulation study: assuming independent censoring can be a serious issue in the case of strong correlation, whereas using the hazard of the subdistribution can be misleading whenever the treatment changes the hazard of only one of the competing events.

The main innovation and the motivation of the present work was to study the operating characteristics of the test by Peto, which is largely used in the medical literature, though almost absent in statistical publications. We aimed at comparing the test by Peto to the most common ones in the statistical literature, i.e. the test on the cause-specific hazard and the test on the hazard of the subdistribution by Gray. These two tests have already been compared head to head previously (see notably [27] and [28]). The main reason for this is that, despite the fact that these two tests address different questions, these are closely linked to each other and in our experience the interest of physicians in a clinical trial is somewhere in-between. Furthermore, to the best of our knowledge, the behavior of these tests in presence of misclassification of the cause of death had never been studied before; we think that the knowledge of such an aspect for the three tests is of primary importance for their practical use.

As our aim was to compare the tests across objectively characterized scenarios, we also investigated how the power and level of the tests could depend on the correlation between times to death from different causes, which has a precise clinical meaning. For example, positive correlation corresponds to comorbidity, which is quite common in advanced diseases. Negative correlation, too, is interesting as this could correspond to the effect of a standard of care therapy with different modalities that impact both disease control and toxicity. In the adjuvant context for lung cancer, for instance, all patients undergo surgery, either segmentectomy, or lobectomy or pneumonectomy: the greater the portion of lung resected, the lower the risk of relapses (and then of CD) but the higher the risk of pulmonary complications (and then of NCD).

## Conclusions

Testing the treatment effect on the cause-specific death rate requires paying attention to the effect on the competing events. We considered three popular tests among several existing ones: a test based on recurrence data proposed by Peto, the cause-specific test and the cumulative incidence test proposed by Gray.

We performed a simulation study in four clinically relevant scenarios, with negatively correlated, uncorrelated and positively correlated event times, and with two censoring proportions in addition to complete data. We also generated recurrence times in order to bring to the fore the effects of classifying the cause of death in different ways. The recurrence times, conditional on the time to cancer deaths, followed a uniform distribution, which we considered a reasonable hypothesis. Further, we compared results to those obtained in the case of a high rate of misclassified causes of death.

All the three tests adequately preserved their nominal size when the treatment was completely ineffective. Gr seemed to be the most reliable in the situation of a therapy that reduced the risk of CD and increased that of NCD, provided that causes of death are correctly recorded; otherwise, it performed substantially worse and Pe should be recommended. In all the other situations Gr had the poorest performances, both in terms of the preservation of the nominal size and in terms of power.

CS should be preferred whenever the treatment is expected to be ineffective against the risk of CD and possibly harmful in terms of NCD. A cancer treatment is required to be effective against the risk of CD but not against that ofNCD. In that case, Pe was comparable to CS, except that CS had very high size for NCD in the presence of a high rate of misrecorded causes of death. In our study, Pe did not outperform its competitors in any situation in which the causes of death were correctly classified, whereas it was often the most reliable when the misclassification rate was high.

No clear pattern linked to the dependence between time variables emerged from our study. Censoring always reduces the rejection probabilities of all the tests, notably under the alternative hypothesis. Consequently, the tests are less and less powerful as censoring increases and their differences are less and less pronounced as well.

In the IALT study, the three tests suggested possible harm due to toxicity; Gr was firmly in favor of a benefit versus the risk of CD, whereas CS and Pe were borderline. We showed how the natural graphical representations for the three tests are the Nelson–Aalen estimate of the cumulative cause-specific hazard, the cumulative yearly rates as estimated by Peto, and the Aalen–Johansen estimate of the cumulative incidence function.

This study is the first to compare the operating characteristics of the log-rank test by Peto to those of the two best established tests in the statistical literature. The method used to simulate the data is innovative in that it takes into account the occurrence of recurrences and, at the same time, it is capable of generating both negatively and positively dependent times. This allowed us to study the effect of the reclassification of the causes of death proposed by Peto, without the requirement of assuming independence between CD and NCD. To keep things simple, we chose not to generate times to death from unknown causes. In such cases, multiple imputations or inverse probability weighting techniques exist (see for instance [35]).

## Abbreviations

CD: Cancer death; CIF: Cumulative incidence function; CS: Cause-specific test; Gr: Gray’s test; IALT: International Adjuvant Lung cancer Trial; NCD: Non-cancer death; Pe: Peto test; Rec: Recurrence.

## Competing interests

The authors declare that they have no competing interests.

## Authors’ contributions

FR and SM conceived the study and developed the simulation model. FR was responsible for the simulation study and drafted the manuscript. FR and SM contributed to the interpretation of results and critically revised the report. Both authors read and approved the final manuscript.

## Pre-publication history

The pre-publication history for this paper can be accessed here:

http://www.biomedcentral.com/1471-2288/14/72/prepub

## Supplementary Material

Additional file 1 **Figures A.1 to A.9 and Tables A.1 to A.8. **Detailed results of the simulation study: Correlation between the simulated event times (Figure A1) and empirical rejection probabilities of the tests in the four scenarios, with and without misclassified causes of death (Figures A2-A9 and Tables A1-A8). Click here for file

## References

[B1] Prentice RL Kalbfleisch JD Peterson AV Flournoy N Farewell VT Breslow NE **The analysis of failure times in the presence of competing risks** Biometrics 1978 34 4 541 554 10.2307/2530374 373811

[B2] Gooley T Leisenring W Crowley J Storer B **Estimation of failure probabilities in the presence of competing risks: new representations of old estimators** Stat Med 1999 30 6 695 705 doi:10.1002/(SICI)1097-02581999033018:6<695::AID-SIM60>3.0.CO;2-O 10204198 10.1002/(sici)1097-0258(19990330)18:6<695::aid-sim60>3.0.co;2-o

[B3] Putter H Fiocco M Geskus RB **Tutorial in biostatistics: competing risks and multi-state models** Stat Med 2007 26 11 2389 430 doi:10.1002/sim.2712 10.1002/sim.2712 17031868

[B4] Allignol A Schumacher M Wanner C Drechsler C Beyersmann J **Understanding competing risks: a simulation point of view** BMC Med Res Methodol 2011 11 1 86 doi:10.1186/1471-2288-11-86 10.1186/1471-2288-11-86 21639902 PMC3135581

[B5] Koller M Raatz H Steyerberg E Wolbers M **Competing risks and the clinical community: irrelevance or ignorance?** Stat Med 2012 31 11–12 1089 1097 doi:10.1002/sim.4384 21953401 10.1002/sim.4384 PMC3575691

[B6] Klein JP Shu YY **Multi-state models for bone marrow transplantation studies** Stat Methods Med Res 2002 11 117 139 doi:10.1191/0962280202sm277ra 10.1191/0962280202sm277ra 12040693

[B7] Lim H Zhang X Dyck R Osgood N **Methods of competing risks analysis of end-stage renal disease and mortality among people with diabetes** BMC Med Res Methodol 2010 10 1 97 doi:10.1186/1471-2288-10-97 10.1186/1471-2288-10-97 20964855 PMC2988010

[B8] Deslandes E Chevret S **Joint modeling of multivariate longitudinal data and the dropout process in a competing risk setting: application to icu data** BMC Med Res Methodol 2010 10 1 69 doi:10.1186/1471-2288-10-69 10.1186/1471-2288-10-69 20670425 PMC2923158

[B9] Chappell R **Competing risk analyses: how are they different and why should you care?** Clin Cancer Res 2012 18 8 2127 2129 doi:10.1158/1078-0432.CCR-12-0455 10.1158/1078-0432.CCR-12-0455 22427347

[B10] Dignam JJ Zhang Q Kocherginsky M **The use and interpretation of competing risks regression models** Clin Cancer Res 2012 18 8 2301 2308 doi:10.1158/1078-0432.CCR-11-2097 10.1158/1078-0432.CCR-11-2097 22282466 PMC3328633

[B11] Rauch G Kieser M Ulrich S Doherty P Rauch B Schneider S Riemer T Senges J **Competing time-to-event endpoints in cardiology trials: a simulation study to illustrate the importance of an adequate statistical analysis** Eur J Prev Cardiol 2014 21 1 74 80 doi:10.1177/2047487312460518 10.1177/2047487312460518 22964966

[B12] Pintilie M Competing Risks: A Practical Perspective 2006 New York: Wiley doi:10.1002/9780470870709

[B13] Gaynor JJ Feuer EJ Tan CC Wu DH Little CR Straus DJ Clarkson BD Brennan MF **On the use of cause-specific failure and conditional failure probabilities: examples from clinical oncology data** J Am Stat Assoc 1993 88 422 400 409 doi:10.1080/01621459.1993.10476289 10.1080/01621459.1993.10476289

[B14] Gray RJ **A class of k-sample tests for comparing the cumulative incidence of a competing risk** Ann Stat 1988 16 3 1141 1154 10.1214/aos/1176350951

[B15] Fine JP Gray RJ **A proportional hazards model for the subdistribution of a competing risk** J Am Stat Assoc 1999 94 446 496 509 10.1080/01621459.1999.10474144

[B16] Early Breast Cancer Trialists’ Collaborative Group Treatment of Early Breast Cancer: Worldwide Evidence 1985–1990, vol. 1 1990 Oxford: Oxford University Press

[B17] Early Breast Cancer Trialists’ Collaborative Group **Effects of radiotherapy and surgery in early breast cancer – an overview of the randomized trials** New Engl J Med 1995 333 22 1444 1456 doi:10.1056/NEJM199511303332202 7477144 10.1056/NEJM199511303332202

[B18] Early Breast Cancer Trialists’ Collaborative Group **Tamoxifen for early breast cancer: an overview of the randomised trials** Lancet 1998 351 9114 1451 1467 doi:10.1016/S0140-67369711423-4 9605801

[B19] Early Breast Cancer Trialists’ Collaborative Group **Effects of chemotherapy and hormonal therapy for early breast cancer on recurrence and 15-year survival: an overview of the randomised trials** Lancet 2005 365 1687 1717 doi:10.1016/S0140-67360566544-0 15894097 10.1016/S0140-6736(05)66544-0

[B20] Bourhis J Overgaard J Audry H Ang KK Saunders M Bernier J Horiot J-C Maître AL Pajak TF Poulsen MG O’Sullivan B Dobrowsky W Hliniak A Skladowski K Hay JH Pinto LH Fallai C Fu KK Sylvester R Pignon J-P **Hyperfractionated or accelerated radiotherapy in head and neck cancer: a meta-analysis** Lancet 2006 368 9538 843 854 doi:10.1016/S0140-67360669121-6 10.1016/S0140-6736(06)69121-6 16950362

[B21] Pignon J Bourhis J Domenge C Designé L **Chemotherapy added to locoregional treatment for head and neck squamous-cell carcinoma: three meta-analyses of updated individual data** Lancet 2000 355 9208 949 955 doi:10.1016/S0140-67360090011-4 10.1016/S0140-6736(00)90011-4 10768432

[B22] Pignon J-P le Maître A Maillard E Bourhis J **Meta-analysis of chemotherapy in head and neck cancer (MACH-NC): an update on 93 randomised trials and 17,346 patients** Radiother Oncol 2009 92 1 4 14 doi:10.1016/j.radonc.2009.04.014 10.1016/j.radonc.2009.04.014 19446902

[B23] Early Breast Cancer Trialists’ Collaborative Group **Effects of radiotherapy and of differences in the extent of surgery for early breast cancer on local recurrence and 15-year survival: an overview of the randomised trials** Lancet 2005 366 9503 2087l doi:10.1016/S0140-67360567887-7 16360786 10.1016/S0140-6736(05)67887-7

[B24] Early Breast Cancer Trialists’ Collaborative Group **Relevance of breast cancer hormone receptors and other factors to the efficacy of adjuvant tamoxifen: patient-level meta-analysis of randomised trials** Lancet 2011 378 9793 771 784 doi:10.1016/S0140-67361160993-8 21802721 10.1016/S0140-6736(11)60993-8 PMC3163848

[B25] Dignam JJ Kocherginsky MN **Choice and interpretation of statistical tests used when competing risks are present** J Clin Oncol 2008 26 24 4027 4034 doi:10.1200/JCO.2007.12.9866 10.1200/JCO.2007.12.9866 18711194 PMC2654314

[B26] Pintilie M **Dealing with competing risks: testing covariates and calculating sample size** Stat Med 2002 21 22 3317 3324 doi:10.1002/sim.1271 10.1002/sim.1271 12407674

[B27] Freidlin B Korn EL **Testing treatment effects in the presence of competing risks** Stat Med 2005 24 11 1703 1712 doi:10.1002/sim.2054 10.1002/sim.2054 15706579

[B28] Williamson PR Kolamunnage-Dona R Tudur Smith C **The influence of competing-risks setting on the choice of hypothesis test for treatment effect** Biostatistics 2007 8 4 689 694 doi:10.1093/biostatistics/kxl040 17151088 10.1093/biostatistics/kxl040

[B29] Ruan PK Gray RJ **A method for analyzing disease-specific mortality with missing cause of death information** Lifetime Data Anal 2006 12 1 35 51 doi:10.1007/s10985-005-7219-2 10.1007/s10985-005-7219-2 16583298

[B30] International Adjuvant Lung Cancer Trial Collaborative Group **Cisplatin-based adjuvant chemotherapy in patients with completely resected non-small-cell lung cancer** New Engl J Med 2004 350 4 351 doi:10.1056/NEJMoa031644 14736927 10.1056/NEJMoa031644

[B31] Arriagada R Dunant A Pignon J-P Bergman B Chabowski M Grunenwald D Kozlowski M Le Péchoux C Pirker R Pinel M-IS Tarayre M Le Chevalier T **Long-Term results of the international adjuvant lung cancer trial evaluating adjuvant Cisplatin-Based chemotherapy in resected lung cancer** J Clin Oncol 2010 28 1 35 42 doi:10.1200/JCO.2009.23.2272 10.1200/JCO.2009.23.2272 19933916

[B32] Nelson W **Theory and applications of hazard plotting for censored failure data** Technometrics 1972 14 4 945 966 doi:10.1080/00401706.1972.10488991 10.1080/00401706.1972.10488991

[B33] Aalen O **Nonparametric inference for a family of counting processes** Ann Stat 1978 6 3 701 726 doi:10.1214/aos/1176344198

[B34] Aalen OO Johansen S **An empirical transition matrix for non-homogeneous markov chains based on censored observations** Scand J Stat 1978 5 3 141 150

[B35] Moreno-Betancur M Latouche A **Regression modeling of the cumulative incidence function with missing causes of failure using pseudo-values** Stat Med 2013 32 18 3206 3223 doi:10.1002/sim.5755 10.1002/sim.5755 23653257

