# Supplementary material for: Testing the treatment effect on competing causes of death in oncology clinical trials
Source: BMC Med Res Methodol. 2014 May 29;14:72. doi: 10.1186/1471-2288-14-72 (PMC4047005; doi:10.1186/1471-2288-14-72)
Supplement: Additional file 1 — Figures A.1 to A.9 and Tables A.1 to A.8. Detailed results of the simulation study: Correlation between the simulated event times (Figure A1) and empirical rejection probabilities of the tests in the four scenarios, with and without misclassified causes of death (Figures A2-A9 and Tables A1-A8). [file 1471-2288-14-72-S1.pdf]

Additional material for "Testing the treatment effect on competing causes of death in oncology clinical trials"

F. Rotolo and S. Michiels

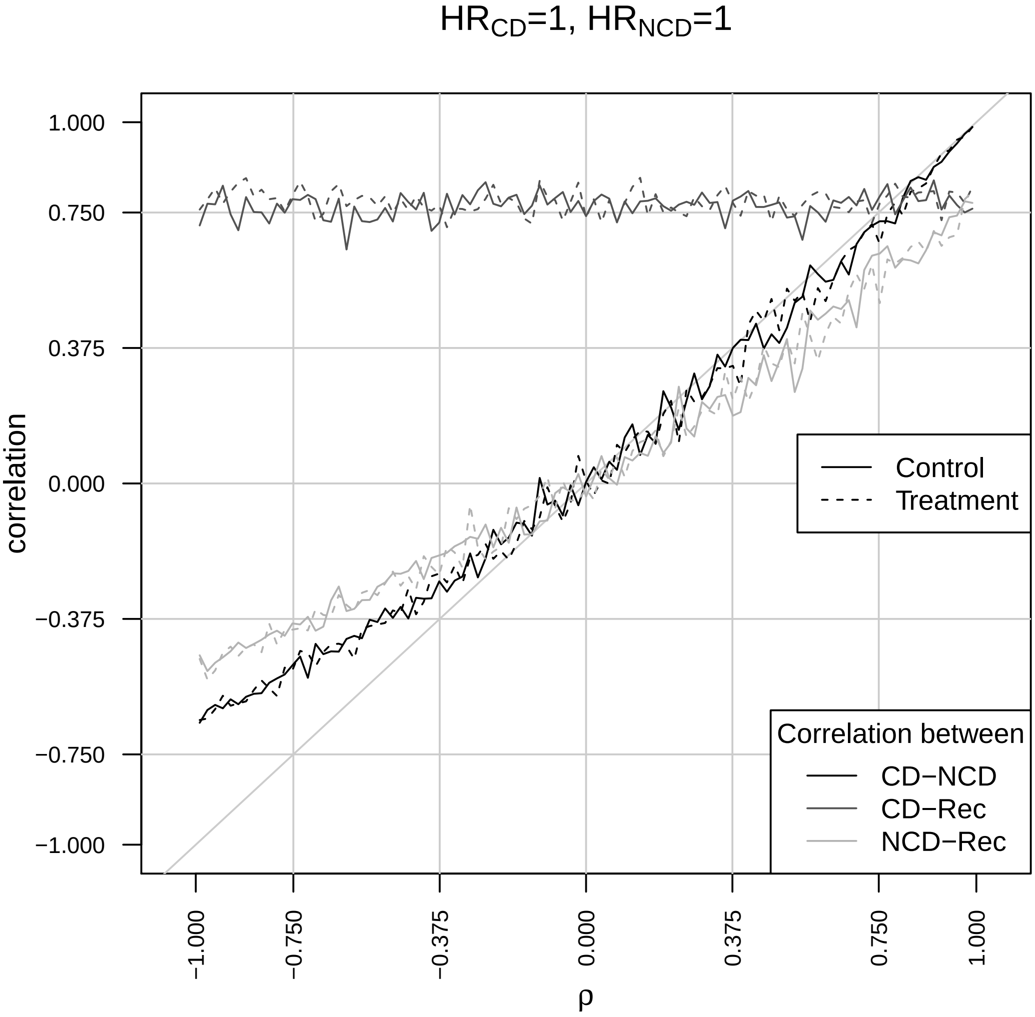

Figure A.1: **Correlations between the three event times.** Pairwise correlations between the three event times in the first scenario considered for the simulation study, as a function of ρ, the correlation between the normal random variables used for data generation. The tick marks correspond to the five values of ρ used for simulations. CD: cancer-deaths; NCD: non-cancer deaths; Rec: recurrences.

Scenario 1: $HR_{\mathsf{CD}} = 1$, $HR_{\mathsf{NCD}} = 1$

Without misclassified causes of death

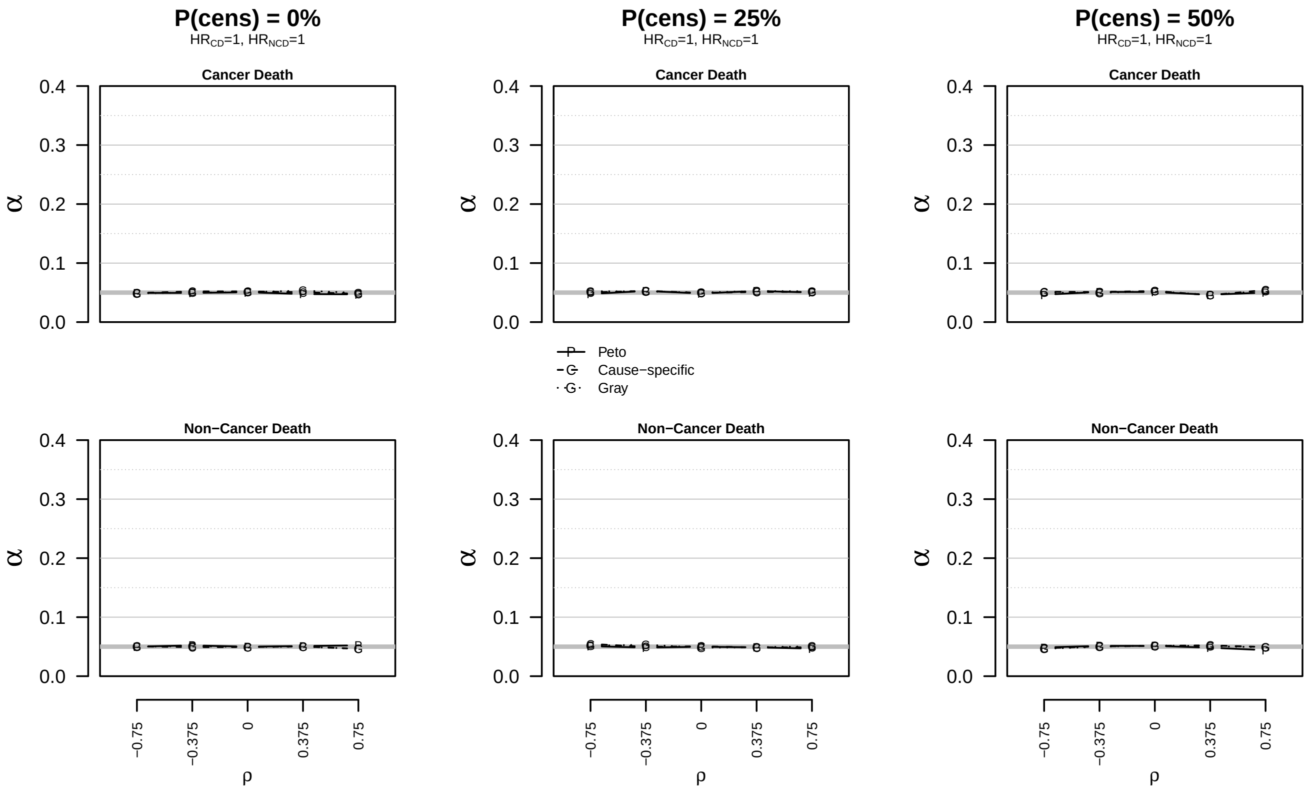

Figure A.2: **Empirical rejection probabilities of the tests in scenario 1 without misclassified causes of death.** Empirical level (α) of the tests for CD and NCD in scenario 1 ($HR_{\mathsf{CD}} = 1$, $HR_{\mathsf{NCD}} = 1$). The plots on the first line concern CD, those on the second line NCD. From left to right, the data are simulated with 0% (P(cens) = 0%), 25% (P(cens) = 25%) and 50% (P(cens) = 50%) censored observations. The grey horizontal line corresponds to the 0.05 level. 10 000 repetitions.

Table A.1: **Empirical rejection probabilities of the tests in scenario 1 without misclassified causes of death.** Empirical level (α) of the tests for CD and NCD in scenario 1 ($HR_{\mathsf{CD}} = 1$, $HR_{\mathsf{NCD}} = 1$). 10 000 repetitions.

	Cancer deaths								
	P(cens) = 0%			P(cens) = 25%			P(cens) = 50%		
ρ	Pe	CS	Gr	Pe	CS	Gr	Pe	CS	Gr
-0.750	0.049	0.049	0.049	0.048	0.050	0.052	0.046	0.051	0.050
-0.375	0.050	0.052	0.050	0.053	0.053	0.052	0.051	0.051	0.050
0.000	0.051	0.052	0.051	0.048	0.049	0.050	0.051	0.053	0.052
0.375	0.048	0.051	0.053	0.053	0.051	0.053	0.046	0.046	0.046
0.750	0.047	0.048	0.049	0.050	0.051	0.052	0.050	0.054	0.053
	Non-cancer deaths								
	P(cens) = 0%			P(cens) = 25%			P(cens) = 50%		
ρ	Pe	CS	Gr	Pe	CS	Gr	Pe	CS	Gr
-0.750	0.050	0.050	0.050	0.051	0.055	0.052	0.049	0.047	0.046
-0.375	0.052	0.049	0.051	0.048	0.050	0.053	0.051	0.051	0.050
0.000	0.050	0.049	0.049	0.050	0.051	0.048	0.051	0.051	0.052
0.375	0.051	0.050	0.050	0.049	0.049	0.049	0.048	0.052	0.051
0.750	0.052	0.046	0.046	0.047	0.049	0.050	0.044	0.049	0.049

Scenario 1: $HR_{\mathsf{CD}} = 1$, $HR_{\mathsf{NCD}} = 1$

With 20% misclassified causes of death

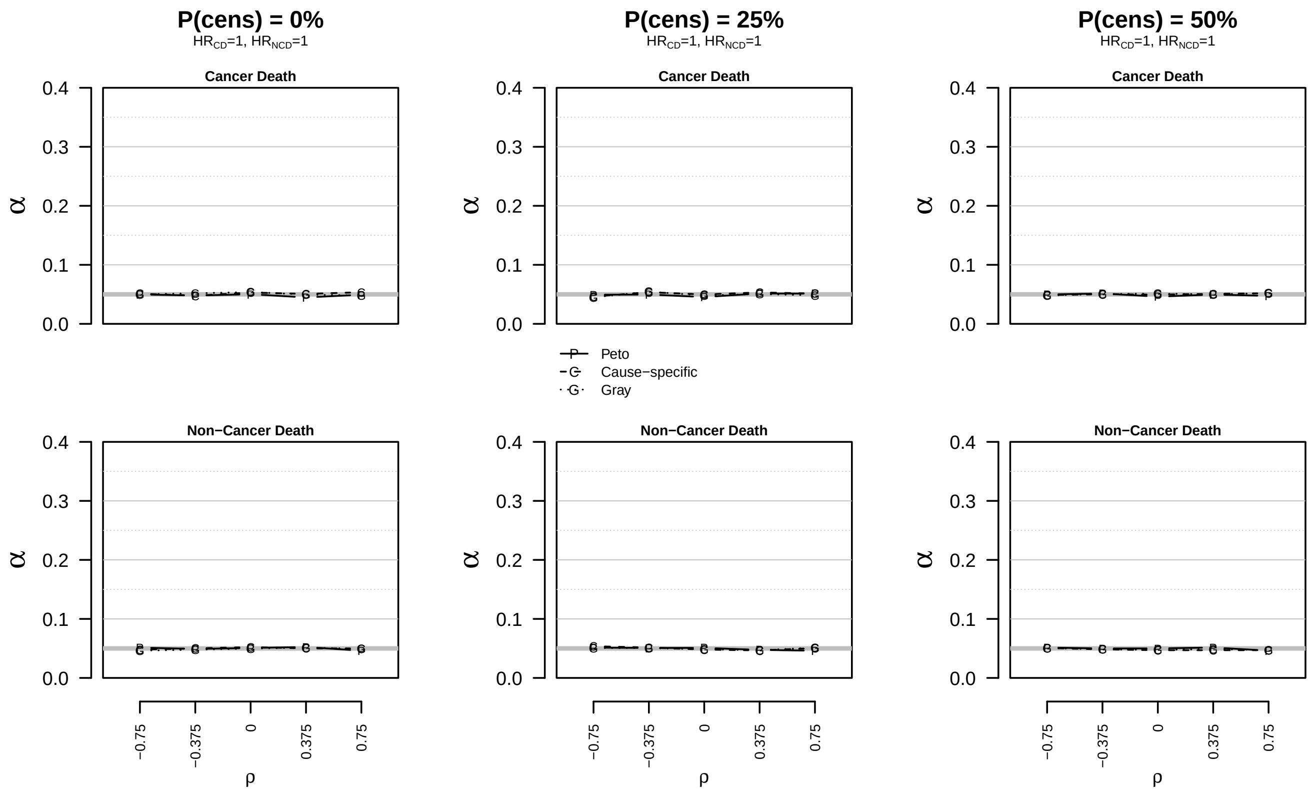

Figure A.3: **Empirical rejection probabilities of the tests in scenario 1 with 20% misclassified causes of death.** Empirical level (α) of the tests for CD and NCD in scenario 1 ($HR_{\mathsf{CD}} = 1$, $HR_{\mathsf{NCD}} = 1$). The plots on the first line concern CD, those on the second line NCD. From left to right, the data are simulated with 0% (P(cens) = 0%), 25% (P(cens) = 25%) and 50% (P(cens) = 50%) censored observations. The grey horizontal line corresponds to the 0.05 level. 10 000 repetitions.

Table A.2: **Empirical rejection probabilities of the tests in scenario 1 with 20% misclassified causes of death.** Empirical level (α) of the tests for CD and NCD in scenario 1 ($HR_{\mathsf{CD}} = 1$, $HR_{\mathsf{NCD}} = 1$). 10 000 repetitions.

	Cancer deaths								
	P(cens) = 0%			P(cens) = 25%			P(cens) = 50%		
ρ	Pe	CS	Gr	Pe	CS	Gr	Pe	CS	Gr
-0.750	0.050	0.051	0.050	0.049	0.046	0.045	0.050	0.049	0.048
-0.375	0.048	0.048	0.052	0.050	0.054	0.054	0.051	0.050	0.051
0.000	0.050	0.053	0.054	0.045	0.050	0.048	0.046	0.050	0.051
0.375	0.045	0.051	0.050	0.052	0.053	0.050	0.050	0.051	0.050
0.750	0.049	0.054	0.048	0.051	0.051	0.048	0.047	0.052	0.052

	Non-cancer deaths								
	P(cens) = 0%			P(cens) = 25%			P(cens) = 50%		
ρ	Pe	CS	Gr	Pe	CS	Gr	Pe	CS	Gr
-0.750	0.051	0.048	0.046	0.051	0.054	0.051	0.052	0.051	0.051
-0.375	0.049	0.050	0.048	0.051	0.052	0.051	0.050	0.048	0.048
0.000	0.051	0.052	0.050	0.051	0.048	0.049	0.050	0.047	0.048
0.375	0.052	0.050	0.051	0.048	0.047	0.047	0.052	0.047	0.049
0.750	0.046	0.050	0.049	0.046	0.050	0.051	0.046	0.047	0.047

Scenario 2: $HR_{\mathsf{CD}} = 1$, $HR_{\mathsf{NCD}} = 1.25$

Without misclassified causes of death

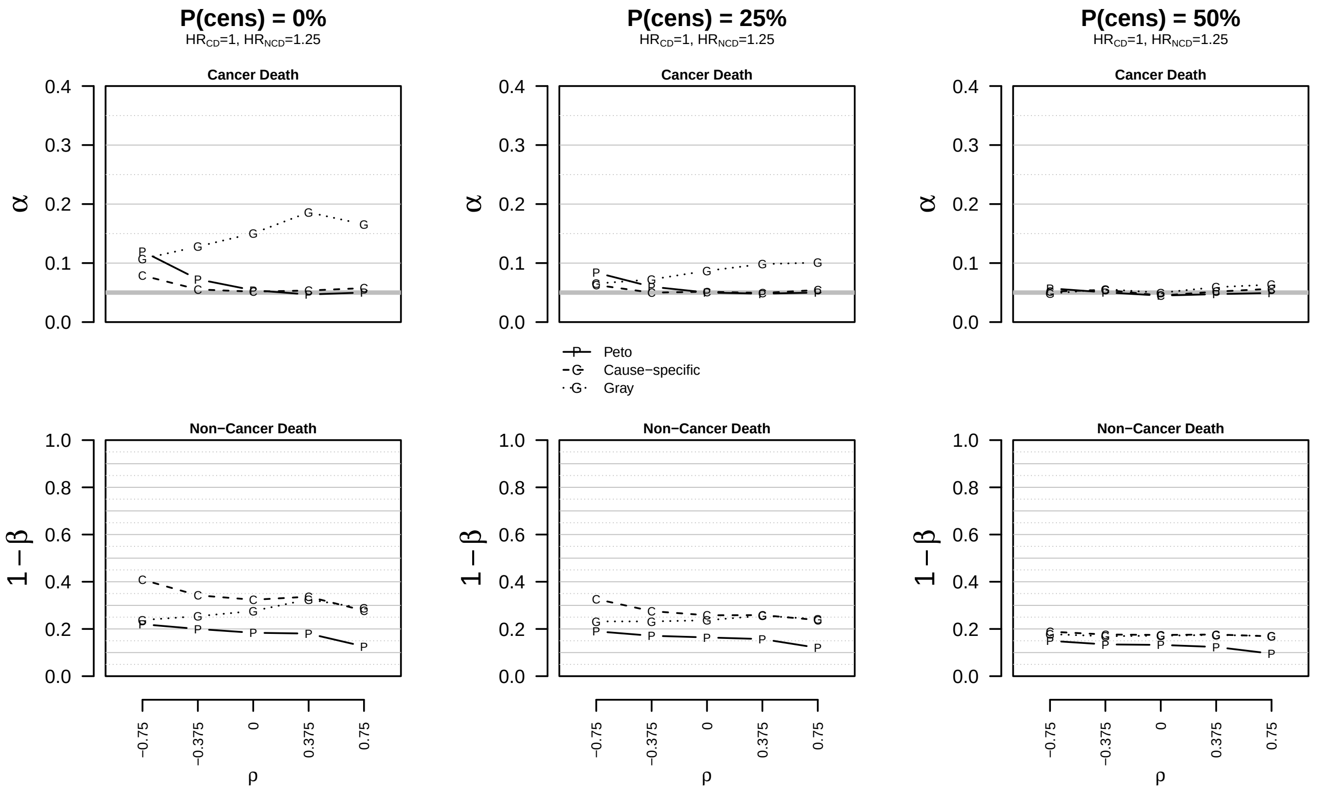

Figure A.4: **Empirical rejection probabilities of the tests in scenario 2 without misclassified causes of death.** Empirical level (α) and power ($1-\beta$) of the tests for CD and NCD in scenario 2 ($HR_{\mathsf{CD}} = 1$, $HR_{\mathsf{NCD}} = 1.25$). The plots on the first line concern CD, those on the second line NCD. From left to right, the data are simulated with 0% (P(cens) = 0%), 25% (P(cens) = 25%) and 50% (P(cens) = 50%) censored observations. The grey horizontal line corresponds to the 0.05 level. 10 000 repetitions.

Table A.3: **Empirical rejection probabilities of the tests in scenario 2 without misclassified causes of death.** Empirical level (α) and power ($1-\beta$) of the tests for CD and NCD in scenario 2 ($HR_{\mathsf{CD}} = 1$, $HR_{\mathsf{NCD}} = 1.25$). 10 000 repetitions.

	Cancer deaths								
	P(cens) = 0%			P(cens) = 25%			P(cens) = 50%		
ρ	Pe	CS	Gr	Pe	CS	Gr	Pe	CS	Gr
-0.750	0.120	0.079	0.107	0.084	0.063	0.065	0.057	0.052	0.048
-0.375	0.073	0.055	0.128	0.060	0.050	0.072	0.050	0.055	0.056
0.000	0.054	0.052	0.150	0.050	0.052	0.087	0.045	0.045	0.050
0.375	0.047	0.054	0.186	0.048	0.049	0.098	0.047	0.052	0.059
0.750	0.050	0.058	0.166	0.050	0.054	0.101	0.049	0.056	0.064
	Non-cancer deaths								
	P(cens) = 0%			P(cens) = 25%			P(cens) = 50%		
ρ	Pe	CS	Gr	Pe	CS	Gr	Pe	CS	Gr
-0.750	0.220	0.409	0.238	0.190	0.327	0.232	0.150	0.189	0.178
-0.375	0.200	0.342	0.254	0.171	0.276	0.232	0.134	0.177	0.169
0.000	0.184	0.324	0.276	0.164	0.258	0.237	0.132	0.175	0.172
0.375	0.180	0.337	0.325	0.157	0.259	0.256	0.124	0.176	0.175
0.750	0.126	0.278	0.287	0.120	0.238	0.242	0.095	0.169	0.170

Scenario 2: $HR_{\mathsf{CD}} = 1$, $HR_{\mathsf{NCD}} = 1.25$

With 20% misclassified causes of death

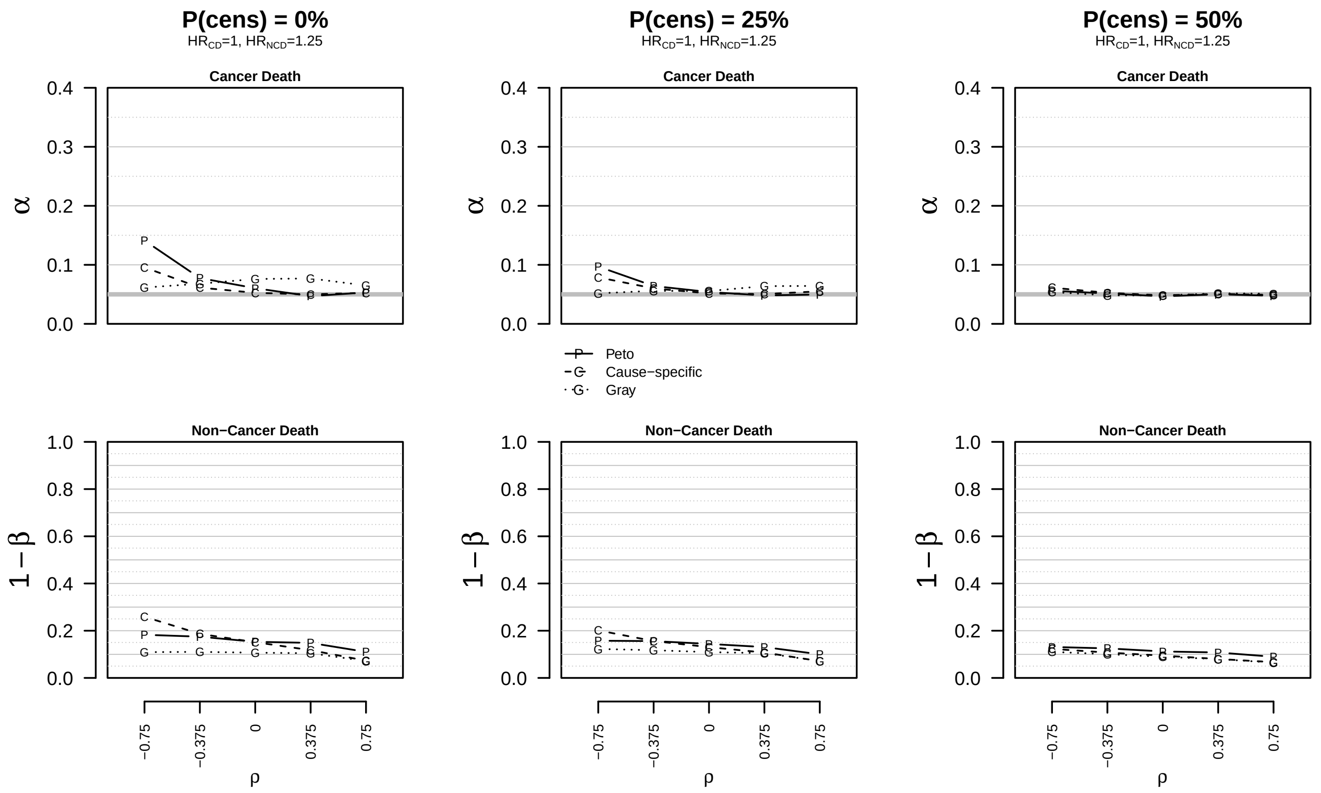

Figure A.5: **Empirical rejection probabilities of the tests in scenario 2 with 20% misclassified causes of death.** Empirical level (α) and power ($1-\beta$) of the tests for CD and NCD in scenario 2 ($HR_{\mathsf{CD}} = 1$, $HR_{\mathsf{NCD}} = 1.25$). The plots on the first line concern CD, those on the second line NCD. From left to right, the data are simulated with 0% (P(cens) = 0%), 25% (P(cens) = 25%) and 50% (P(cens) = 50%) censored observations. The grey horizontal line corresponds to the 0.05 level. 10 000 repetitions.

Table A.4: **Empirical rejection probabilities of the tests in scenario 2 with 20% misclassified causes of death.** Empirical level (α) and power ($1-\beta$) of the tests for CD and NCD in scenario 2 ($HR_{\mathsf{CD}} = 1$, $HR_{\mathsf{NCD}} = 1.25$). 10 000 repetitions.

	Cancer deaths								
	P(cens) = 0%			P(cens) = 25%			P(cens) = 50%		
ρ	Pe	CS	Gr	Pe	CS	Gr	Pe	CS	Gr
-0.750	0.141	0.096	0.062	0.097	0.079	0.052	0.056	0.061	0.054
-0.375	0.078	0.061	0.068	0.064	0.060	0.056	0.052	0.053	0.048
0.000	0.060	0.053	0.076	0.054	0.052	0.056	0.047	0.048	0.048
0.375	0.047	0.050	0.077	0.048	0.050	0.064	0.050	0.051	0.052
0.750	0.053	0.052	0.065	0.050	0.055	0.064	0.048	0.049	0.051
	Non-cancer deaths								
	P(cens) = 0%			P(cens) = 25%			P(cens) = 50%		
ρ	Pe	CS	Gr	Pe	CS	Gr	Pe	CS	Gr
-0.750	0.183	0.259	0.109	0.158	0.201	0.123	0.131	0.123	0.110
-0.375	0.175	0.187	0.110	0.156	0.156	0.117	0.125	0.107	0.101
0.000	0.153	0.151	0.107	0.144	0.130	0.109	0.112	0.095	0.090
0.375	0.148	0.118	0.105	0.131	0.108	0.105	0.108	0.080	0.080
0.750	0.110	0.073	0.072	0.100	0.071	0.071	0.090	0.067	0.065

Scenario 3: $HR_{\mathsf{CD}} = 0.8$, $HR_{\mathsf{NCD}} = 1$

Without misclassified causes of death

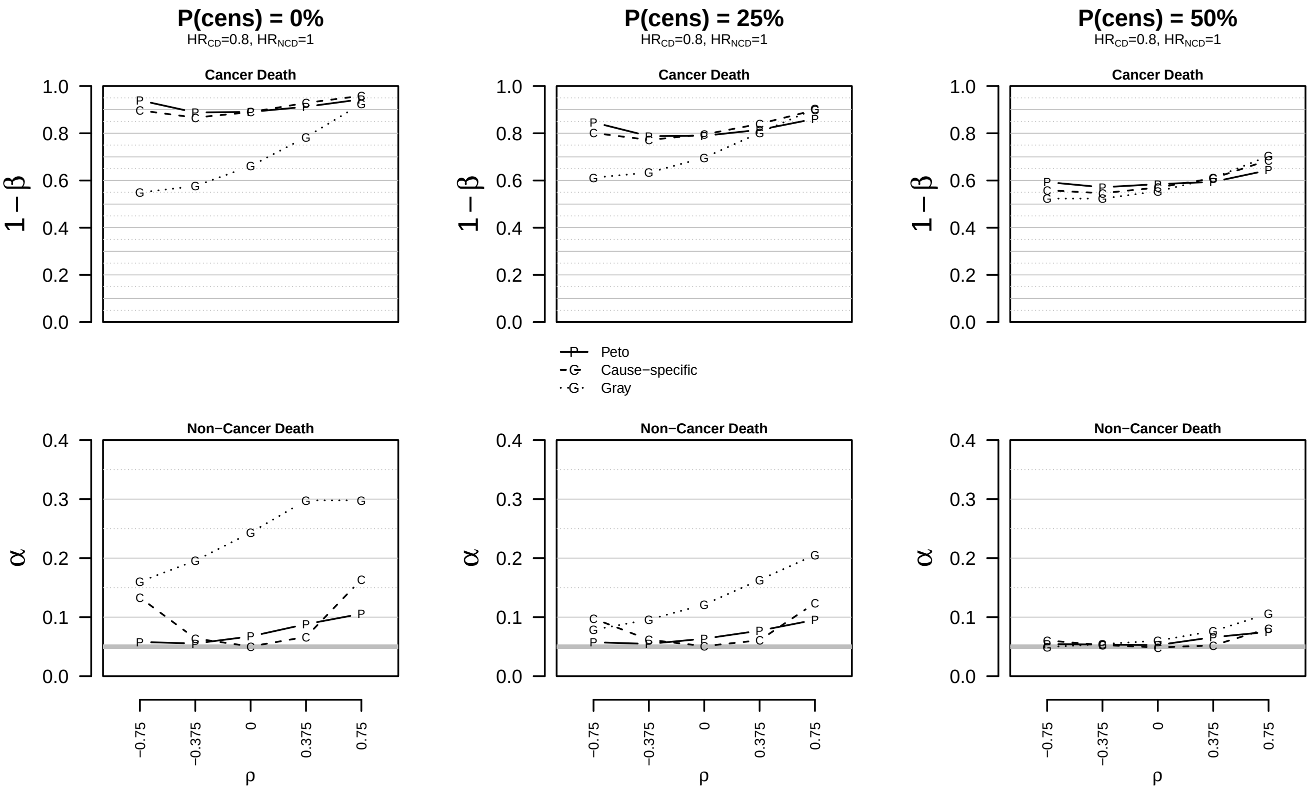

Figure A.6: **Empirical rejection probabilities of the tests in scenario 3 without misclassified causes of death.** Empirical power $(1-\beta)$ ans level (α) of the tests for CD and NCD in scenario 3 ($HR_{\mathsf{CD}} = 0.8$, $HR_{\mathsf{NCD}} = 1$). The plots on the first line concern CD, those on the second line NCD. From left to right, the data are simulated with 0% (P(cens) = 0%), 25% (P(cens) = 25%) and 50% (P(cens) = 50%) censored observations. The grey horizontal line corresponds to the 0.05 level. 10 000 repetitions.

Table A.5: **Empirical rejection probabilities of the tests in scenario 3 without misclassified causes of death.** Empirical power $(1-\beta)$ ans level (α) of the tests for CD and NCD in scenario 3 ($HR_{\mathsf{CD}} = 0.8$, $HR_{\mathsf{NCD}} = 1$). 10 000 repetitions.

	Cancer deaths								
	P(cens) = 0%			P(cens) = 25%			P(cens) = 50%		
ρ	Pe	CS	Gr	Pe	CS	Gr	Pe	CS	Gr
-0.750	0.940	0.897	0.549	0.846	0.801	0.612	0.593	0.559	0.523
-0.375	0.888	0.866	0.576	0.788	0.772	0.633	0.571	0.546	0.523
0.000	0.890	0.890	0.661	0.789	0.795	0.696	0.584	0.570	0.554
0.375	0.912	0.928	0.784	0.815	0.840	0.802	0.594	0.609	0.610
0.750	0.944	0.959	0.924	0.861	0.898	0.904	0.644	0.686	0.704
	Non-cancer deaths								
	P(cens) = 0%			P(cens) = 25%			P(cens) = 50%		
ρ	Pe	CS	Gr	Pe	CS	Gr	Pe	CS	Gr
-0.750	0.058	0.133	0.160	0.058	0.098	0.079	0.054	0.060	0.049
-0.375	0.055	0.064	0.196	0.055	0.062	0.096	0.054	0.053	0.054
0.000	0.067	0.050	0.243	0.064	0.051	0.121	0.053	0.049	0.060
0.375	0.088	0.066	0.298	0.077	0.061	0.163	0.066	0.052	0.076
0.750	0.106	0.164	0.298	0.095	0.124	0.205	0.075	0.081	0.106

Scenario 3: $HR_{\text{CD}} = 0.8$, $HR_{\text{NCD}} = 1$

With 20% misclassified causes of death

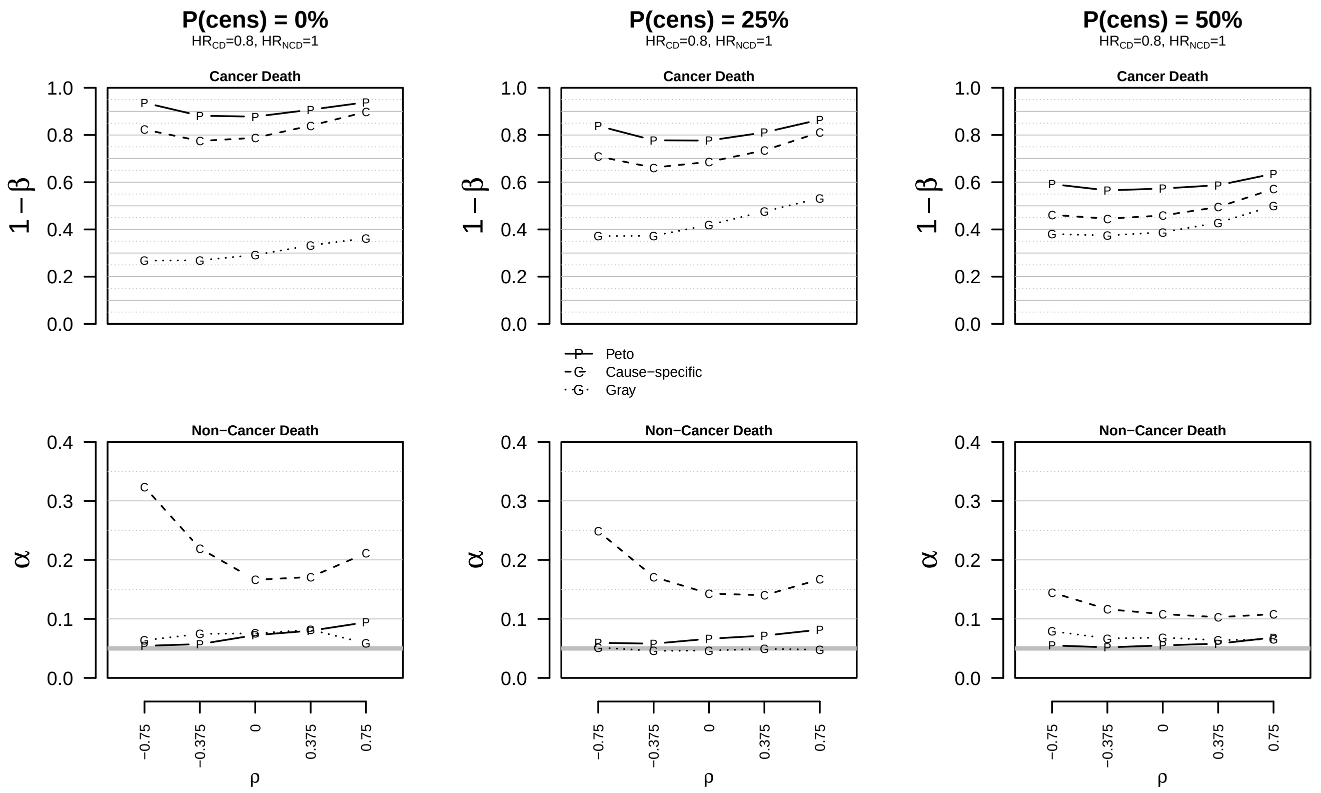

Figure A.7: **Empirical rejection probabilities of the tests in scenario 3 with 20% misclassified causes of death.** Empirical power $(1-\beta)$ ans level (α) of the tests for CD and NCD in scenario 3 ($HR_{\text{CD}} = 0.8$, $HR_{\text{NCD}} = 1$). The plots on the first line concern CD, those on the second line NCD. From left to right, the data are simulated with 0% (P(cens) = 0%), 25% (P(cens) = 25%) and 50% (P(cens) = 50%) censored observations. The grey horizontal line corresponds to the 0.05 level. 10 000 repetitions.

Table A.6: **Empirical rejection probabilities of the tests in scenario 3 with 20% misclassified causes of death.** Empirical power $(1-\beta)$ ans level (α) of the tests for CD and NCD in scenario 3 ($HR_{\text{CD}} = 0.8$, $HR_{\text{NCD}} = 1$). 10 000 repetitions.

	Cancer deaths								
	P(cens) = 0%			P(cens) = 25%			P(cens) = 50%		
ρ	Pe	CS	Gr	Pe	CS	Gr	Pe	CS	Gr
-0.750	0.937	0.823	0.268	0.839	0.709	0.372	0.592	0.462	0.380
-0.375	0.882	0.775	0.269	0.778	0.662	0.373	0.565	0.446	0.374
0.000	0.878	0.788	0.292	0.776	0.686	0.419	0.573	0.459	0.388
0.375	0.907	0.840	0.333	0.811	0.735	0.476	0.587	0.495	0.428
0.750	0.939	0.898	0.363	0.865	0.811	0.532	0.636	0.571	0.500
	Non-cancer deaths								
	P(cens) = 0%			P(cens) = 25%			P(cens) = 50%		
ρ	Pe	CS	Gr	Pe	CS	Gr	Pe	CS	Gr
-0.750	0.054	0.324	0.064	0.060	0.248	0.052	0.055	0.145	0.079
-0.375	0.057	0.220	0.075	0.058	0.171	0.046	0.052	0.116	0.067
0.000	0.072	0.166	0.076	0.066	0.143	0.046	0.055	0.108	0.068
0.375	0.080	0.171	0.082	0.072	0.140	0.049	0.058	0.103	0.064
0.750	0.094	0.211	0.059	0.082	0.167	0.048	0.068	0.108	0.066

Scenario 4: $HR_{\mathsf{CD}} = 0.8$, $HR_{\mathsf{NCD}} = 1.25$

Without misclassified causes of death

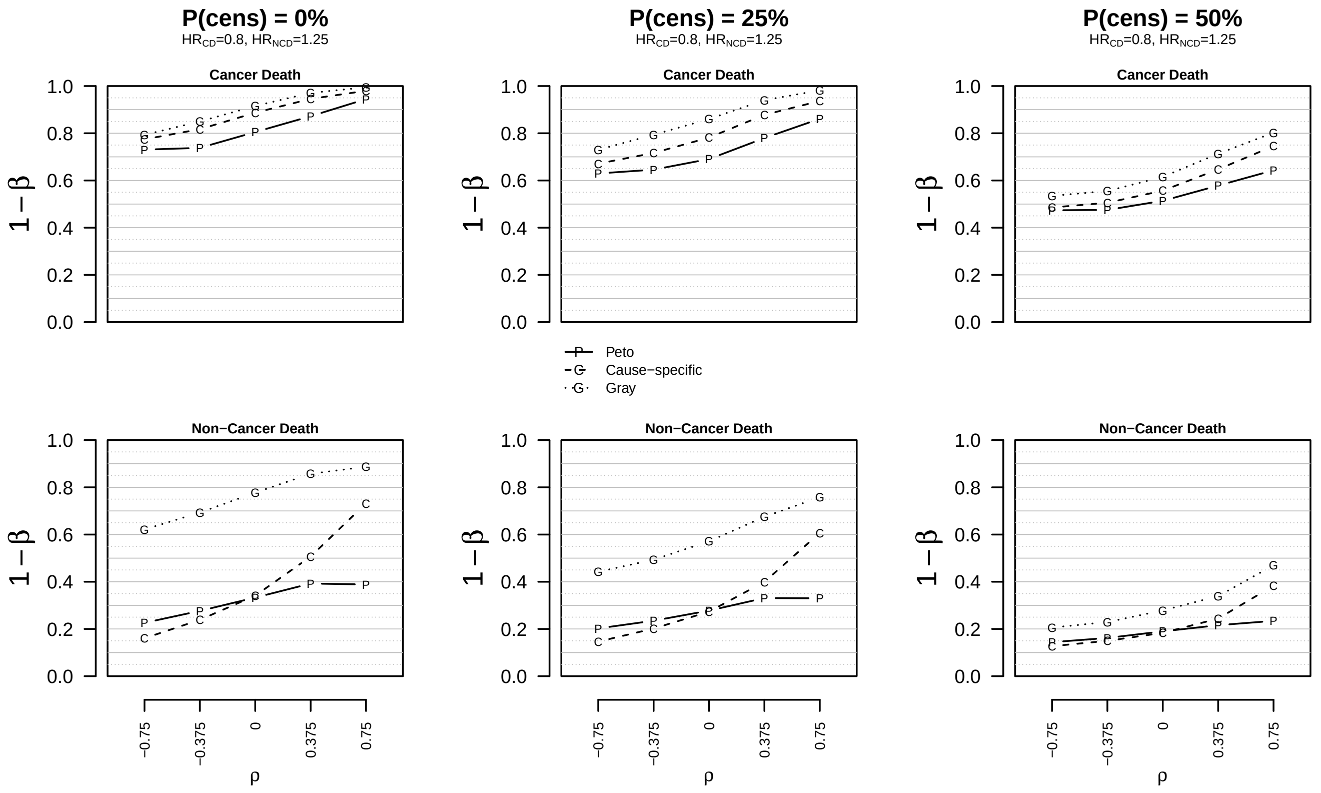

Figure A.8: **Empirical rejection probabilities of the tests in scenario 4 without misclassified causes of death.** Empirical power $(1-\beta)$ of the tests for CD and NCD in scenario 4 ($HR_{\mathsf{CD}} = 0.8$, $HR_{\mathsf{NCD}} = 1.25$). The plots on the first line concern CD, those on the second line NCD. From left to right, the data are simulated with 0% (P(cens) = 0%), 25% (P(cens) = 25%) and 50% (P(cens) = 50%) censored observations. 10 000 repetitions.

Table A.7: **Empirical rejection probabilities of the tests in scenario 4 without misclassified causes of death.** Empirical power $(1-\beta)$ of the tests for CD and NCD in scenario 4 ($HR_{\mathsf{CD}} = 0.8$, $HR_{\mathsf{NCD}} = 1.25$). 10 000 repetitions.

	Cancer deaths								
	P(cens) = 0%			P(cens) = 25%			P(cens) = 50%		
ρ	Pe	CS	Gr	Pe	CS	Gr	Pe	CS	Gr
-0.750	0.731	0.774	0.793	0.630	0.671	0.730	0.474	0.486	0.535
-0.375	0.738	0.817	0.850	0.646	0.716	0.794	0.475	0.505	0.555
0.000	0.805	0.887	0.916	0.691	0.782	0.860	0.514	0.557	0.615
0.375	0.872	0.946	0.971	0.780	0.878	0.940	0.578	0.647	0.713
0.750	0.945	0.980	0.995	0.861	0.937	0.982	0.643	0.746	0.802
	Non-cancer deaths								
	P(cens) = 0%			P(cens) = 25%			P(cens) = 50%		
ρ	Pe	CS	Gr	Pe	CS	Gr	Pe	CS	Gr
-0.750	0.226	0.161	0.621	0.202	0.146	0.442	0.144	0.127	0.206
-0.375	0.276	0.239	0.693	0.235	0.202	0.494	0.162	0.150	0.229
0.000	0.333	0.341	0.778	0.278	0.273	0.571	0.190	0.184	0.279
0.375	0.392	0.507	0.857	0.331	0.399	0.675	0.216	0.244	0.339
0.750	0.389	0.732	0.887	0.330	0.607	0.758	0.234	0.383	0.470

Scenario 4: $HR_{\mathsf{CD}} = 0.8$, $HR_{\mathsf{NCD}} = 1.25$

With 20% misclassified causes of death

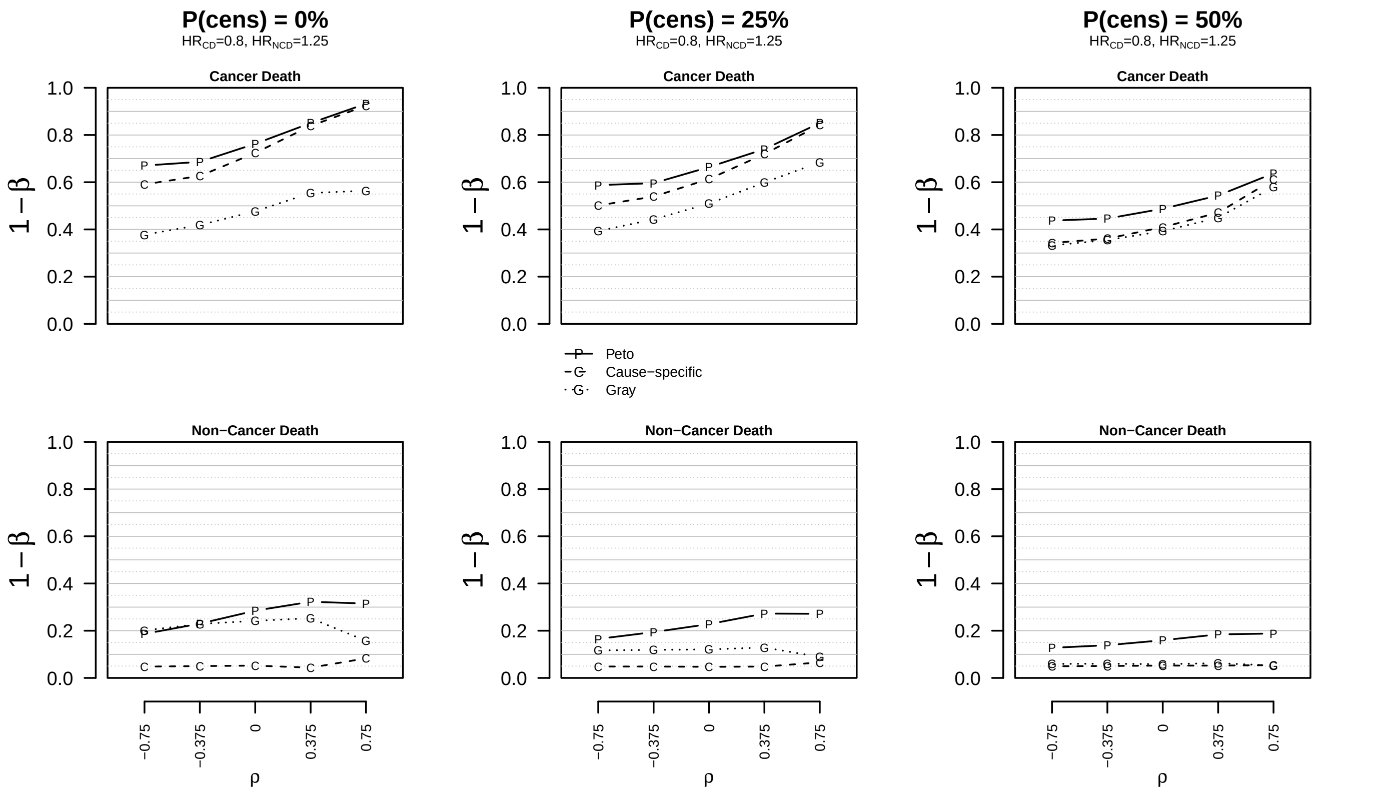

Figure A.9: **Empirical rejection probabilities of the tests in scenario 4 with 20% misclassified causes of death.** Empirical power $(1 - \beta)$ of the tests for CD and NCD in scenario 4 ($HR_{\mathsf{CD}} = 0.8$, $HR_{\mathsf{NCD}} = 1.25$). The plots on the first line concern CD, those on the second line NCD. From left to right, the data are simulated with 0% (P(cens) = 0%), 25% (P(cens) = 25%) and 50% (P(cens) = 50%) censored observations. 10 000 repetitions.

Table A.8: **Empirical rejection probabilities of the tests in scenario 4 with 20% misclassified causes of death.** Empirical power $(1 - \beta)$ of the tests for CD and NCD in scenario 4 ($HR_{\mathsf{CD}} = 0.8$, $HR_{\mathsf{NCD}} = 1.25$). 10 000 repetitions.

	Cancer deaths								
	P(cens) = 0%			P(cens) = 25%			P(cens) = 50%		
ρ	Pe	CS	Gr	Pe	CS	Gr	Pe	CS	Gr
-0.750	0.670	0.591	0.378	0.588	0.501	0.393	0.438	0.343	0.332
-0.375	0.686	0.626	0.419	0.596	0.539	0.443	0.446	0.361	0.355
0.000	0.763	0.724	0.477	0.664	0.614	0.510	0.488	0.410	0.393
0.375	0.850	0.838	0.554	0.740	0.719	0.599	0.544	0.471	0.450
0.750	0.932	0.924	0.564	0.851	0.843	0.685	0.638	0.611	0.580
	Non-cancer deaths								
	P(cens) = 0%			P(cens) = 25%			P(cens) = 50%		
ρ	Pe	CS	Gr	Pe	CS	Gr	Pe	CS	Gr
-0.750	0.670	0.591	0.378	0.588	0.501	0.393	0.438	0.343	0.332
-0.375	0.686	0.626	0.419	0.596	0.539	0.443	0.446	0.361	0.355
0.000	0.763	0.724	0.477	0.664	0.614	0.510	0.488	0.410	0.393
0.375	0.850	0.838	0.554	0.740	0.719	0.599	0.544	0.471	0.450
0.750	0.932	0.924	0.564	0.851	0.843	0.685	0.638	0.611	0.580
